# During the summer 2009 outbreak of "swine flu" in Scotland what respiratory pathogens were diagnosed as H1N1/2009?

**DOI:** 10.1186/1471-2334-11-192

**Published:** 2011-07-13

**Authors:** Rory N Gunson, William F Carman

**Affiliations:** 1 West Of Scotland Specialist Virology Centre, A Member of the UK Clinical Virology Network, Gartnavel General Hospital, Great Western Road, Glasgow, G12 OYN, UK

## Abstract

**Background:**

During the April-July 2009 outbreak of H1N1/2009 in scotland the West of Scotland Specialist Virology Centre (WoSSVC) in Glasgow tested > 16 000 clinical samples for H1N1/2009. Most were from patients clinically diagnosed with H1N1/2009. Out of these, 9% were positive. This study sought to determine what respiratory pathogens were misdiagnosed as cases of H1N1/2009 during this time.

**Methods:**

We examined the results from 3247 samples which were sent to the laboratory during April-July 2009. All were from patients clinically diagnosed as having H1N1/2009 (based on accepted criteria) and all were given a full respiratory screen using real time reverse transcriptase polymerase chain reaction (rtRT-PCR) assays.

**Results:**

In total, respiratory pathogens were detected in 27.9% (95% confidence interval, 26.3-29.5%) of the samples submitted. Numerous pathogens were detected, the most common of which were rhinovirus (8.9% (95% confidence interval, 7.9-9.9%)), parainfluenza 1 (1.9% (95% confidence interval, 1.4-2.4%)) and 3 (4.1% (95% confidence interval, 3.3-4.9%)), and adenovirus ((3.5% (95% confidence interval, 2.9-4.2%)).

**Conclusions:**

This study highlights the problems of using a clinical algorithm to detect H1N1/2009. Clinicians frequently misdiagnosed common respiratory pathogens as H1N1/2009 during the spring/summer outbreak in Scotland. Many undesirable consequences would have resulted, relating to treatment, infection control, and public health surveillance.

## Background

On April 15^th ^and 17^th ^2009, a novel swine-lineage influenza A (H1N1/2009) infection was reported to the World Health Organisation (WHO) by the Centers for Disease Control and Prevention (CDC) in Atlanta. The virus was detected in two children from adjacent counties in southern California presenting with febrile respiratory illness [1,2]. These cases were not epidemiologically linked and neither child had exposure to swine. Subsequent phylogenetic characterisation of H1N1/2009 from the U.S. index case (A/California/04/2009) showed that the virus had a unique genome composition that had not been previously identified. Six genes (PB2, PB1, PA, HA, NP, and NS) were similar to viruses previously identified in triple-reassortant swine influenza viruses in North American pigs. The remaining two genes (NA and M) were derived from Eurasian swine influenza viruses. This particular gene constellation had never been previously identified in humans or other reservoirs.

Following the original identification of Influenza A/H1N1/2009 in the United States, sustained human-to-human transmission was seen in other countries, and on June 11, 2009, the WHO declared that the virus was responsible for the first influenza pandemic of the 21st century [3].

The first cases of H1N1/2009 in Scotland were detected at the end of April 2009 in a couple returning from their honeymoon in Mexico [4,5]. The initial public health response to the outbreak was a containment exercise aimed at preventing the spread of infection, detecting cases and taking action to prevent these cases from infecting others [6]. The exercise was initially based on clinical and epidemiological criteria (table 1). Patients who met these criteria were immediately given treatment and isolated while a rapid real time reverse transcriptase polymerase chain reaction (rtRT-PCR) for H1N1/2009 was initiated. Contact tracing was undertaken in order to treat those who had been in contact with confirmed cases. Soon after the first detections, person to person transmission was confirmed as having occurred in Scotland. Consequently, the epidemiological criteria were no longer useful and the containment exercise was then based on clinical criteria only. Testing continued to be carried out during this period.

**Table 1:** The clinical and epidemiological criteria for the screening and testing of H1N1/2009 cases

Clinical Criteria	Epidemiological criteria
Fever ***or ***history of fever **and **flu-like illness **or **severe life threatening illness suggestive of an infectious process	Onset of illness within 7 days of travelling to an area where sustained transmission of H1N1/2009 is occurring Contacts with a probable/confirmed case of H1N1/2009

**Figure 1:**
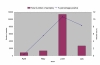
**H1N1 outbreak in the spring/summer 2009**.

During the outbreak period (April-July 2009) the West of Scotland Specialist Virology Centre (WoSSVC) tested 16 264 clinical samples for H1N1/2009 (Figure 1). Of these, only 1516 were positive (9% overall for the period of April-July; range 5-10% per month). Consequently, the clinical diagnosis was found to be wrong in the majority of cases.

A large number of viral infections, drugs and other diseases can cause disease presentations similar to those presented in Table 1. This is especially true for respiratory pathogens. The present study sought to determine what respiratory pathogens were diagnosed as cases of H1N1/2009 during the containment phase.

## Methods

### Background to the respiratory service in place during April-July 2009

During April-July 2009 all respiratory samples submitted from patients with a clinical diagnosis of H1N1/2009 (as stated on the specimen request form), were initially tested using a universal influenza A real time rtRT-PCR assay and a H1N1/2009 specific real time rtRT-PCR assay [7].

If the sample was negative on these tests and was from a hospitalised patient, a patient deemed at risk of severe respiratory infection (e.g. an immunocompromised or a pregnant patient), or a patient attending GP services taking part in local or national surveillance schemes, a full respiratory screen was carried out (see below for details regarding the full respiratory screen). Note that this information was derived from the sample request form.

With regard to (i) samples from GP services not taking part in the surveillance schemes mentioned above, (ii) follow-up samples from known H1N1/2009 positive patients, and (iii) samples from patients with no clinical/setting details, only the initial screening test was conducted.

### Samples

To determine which respiratory pathogens had been misdiagnosed as H1N1/2009, we examined the results of 3247 samples which had been given a full respiratory screen during the period April-July 2009.

All had been clinically diagnosed as having H1N1/2009, and all were found to be H1N1/2009 negative. The samples submitted included gargles, throat swabs, nasal swabs, nasopharyngeal aspirates, sputums, and endotracheal secretions. The number collected each month and the age of the patients are shown in Table 2. Binomial 95% confidence intervals are shown for each age group.

**Table 2:** Patient groups per month of the 3247 samples

	April		May		June		July		Total	
	**Number**	**% (95% confidence interval)**	**Number**	**%(95% confidence interval)**	**Number**	**% (95% confidence interval)**	**Number**	**% (95% confidence interval)**	**Number**	**% (95% confidence interval)**

< 1 yrs	101	16.7 (13.8-19.9%)	78	12.1 (9.7-14.8%)	91	11.8 (9.6-14.2%)	124	10.1 (8.5-11.9%)	394	12.1 (11-13.3%)

1-5 yrs	104	17.2 (14.2-20.4%)	95	14.7 (12-17.7%)	129	16.7 (14-19.5%)	143	11.7 (9.9-13.6%)	471	14.5 (13.3-15.7%)

6-16 yrs	49	8.1 (6-10.6%)	50	7.7 (5.8-10.1%)	90	11.6 (9.5-14.1%)	113	9.2 (7.7-11%)	302	9.3 (8.3-10.4%)

17-64 yrs	266	44 (40-48%)	315	48.7 (44.7-52.6 %)	378	48.8 (45-52.4%)	693	56.5 (53.7-59%)	1652	50.9 (49.1-52.6%)

> 65 yrs	84	13.9 (11.2-16.4%)	109	16.8 (14-20%)	87	11.2 (9.1-13.7%)	148	12.1 (10.3-14%)	428	13.2 (12-14.4%)

Total	605		647		774		1226		3247	

### Laboratory Methods

Total nucleic acid was extracted from respiratory specimens using QIAamp Viral RNA kit (Qiagen, Crawley, United Kingdom) according to the manufacturer's instructions.

Real-time RT-PCR was carried out in order to detect influenza A (a generic assay and a H1N1/2009 specific assay [7]), B and C, RSV, rhinovirus, parainfluenza 1-4, human metapneumovirus, coronavirus (229E, NL63, HKU1 and OC43), adenovirus, and *Mycoplasma pneumoniae*.

The oligonucleotide primers and probe (TIB-MOLBIOL, Berlin, Germany) are outlined in Table 3. The primers and probes for the influenza A generic assay and the H1N1/2009 specific assay are described elsewhere [7]. These assays have been developed by the WOSSVC and used as the frontline test for respiratory samples since 2005. All assays have been shown to be sensitive and specific by in-house development procedures and via participation in numerous external quality assessment schemes (EQA), including those provided by the WHO, the Health Protection Agency (HPA), and Quality Controls for Molecular Diagnostics (QCMD).

**Table 3:** Primer and probe sequences for the respiratory multiplex rtPCR

Multiplex	Virus Target	Target Gene	Forward primer sequence	Reverse primer sequence	Probe sequence
1	Influenza B	NS	ATGATCTTACAGTGGAGGATGAAGAA	CGAATTGGCTTTGRATGTCCTT	**CY5-**ATGGCCATCGGATCCTCAAYTCACTCT-**BHQ**
	
	Influenza C	Matrix	GGCAAGCGACATGCTGAAYA	TCCAGCTGCYTTCATTTGCTTT	**VIC**-CTCTTCCTTCTGATTTTTTCAAA-**MGBNFQ**

2	Mycoplasma pneumoniae	Cytadhesin P1 (P1) gene	AAGCAGGAGTGACGGAAACAC	CACCACATCATTCCCCGTATT	**CY5-**CTCCACCAACAACCTCGCGCCTA-BHQ
	
	Human metapneumovirus A	Fusion	GCYGTYAGCTTCAGTCAATTCAA	TCCAGCATTGTCTGAAAATTGC	**VIC**-CAACATTTAGAAACCTTCT-**MGBNFQ**
	
	Human metapneumovirus B	Fusion	GCYGTYAGCTTCAGTCAATTCAA (Common with A)	GTTATCCCTGCATTGTCTGAAAACT	**VIC**-CGCACAACATTTAGGAATCTTCT-**MGBNFQ**
	
	Parainfluenza virus 1	HN	GTGATTTAAACCCGGTAATTTCTCA	CCTTGTTCCTGCAGCTATTACAGA	**FAM**-ACCTATGACATCAACGAC-**MGBNFQ**

3	Parainfluenza virus 2	HN	ATGAAAACCATTTACCTAAGTGATGGA	CCTCCYGGTATRGCAGTGACTGAAC	**VIC**-TCAATCGCAAAAGC-MGBNFQ
	
	Parainfluenza virus 3	HN	CCAGGGATATAYTAYAAAGGCAAAA	CCGGGRCACCCAGTTGTG	**FAM**-TGGRTGTTCAAGACCTCCATAYCCGAGAAA-**BHQ**
	
	Parainfluenza virus 4	Fusion	CAGAYAACATCAATCGCCTTACAAA	TGTACCTATGACTGCCCCAAARA	**CY5**-CCMATCACAAGCTCAGAAATYCAAAGTCGT-**BHQ3A**

4	Human coronavirus 229E	Nucleocapsid	CAGTCAAATGGGCTGATGCA	AAAGGGCTATAAAGAGAATAAGGTATTCT	**FAM**-CCCTGACGACCACGTTGTGGTTCA- **BHQ**
	
	Human coronavirus OC43	Nucleocapsid	CGATGAGGCTATTCCGACTAGGT	CCTTCCTGAGCCTTCAATATAGTAACC	**CY5**-TCCGCCTGGCACGGTACTCCCT- **BHQ**
	
	Human coronavirus NL63	1a gene	ACGTACTTCTATTATGAAGCATGATATTAA	AGCAGATCTAATGTTATACTTAAAACTACG	**VIC**-ATTGCCAAGGCTCCTAAACGTACAGGTGTT-**TAMRA**

5	Respiratory syncitial virus A	NP	AGATCAACTTCTGTCATCCAGCAA	TTCTGCACATCATAATTAGGAG	**FAM**-CACCATCCAACGGAGCACAGGAGAT-**BHQ**
	
	Respiratory syncitial virus B	NP	AAGATGCAAATCATAAATTCACAGGA	TGATATCCAGCATCTTTAAGTA	**FAM**-TTTCCCTTCCTAACCTGGACATA-**BHQ**
	
	Rhinovirus	5'-UTR	TGGACAGGGTGTGAAGAGC	CAAAGTAGTCGGTCCCATCC	**VIC**-TCCTCCGGCCCCTGAATG-**TAMRA**
	
	Adenovirus	Matrix	GCCACGGTGGGGTTTCTAAACTT	GCCCCAGTGGTCTTACATGCACATC	**CY5**-TGCACCAGACCCGGGCTCAGGTACTCCGA-**BHQ**

All assays used the primers at a final concentration of 0.4 μM and the probe at 0.2 μM in a 15 μl reaction volume. One-step rtRT-PCR was performed on 6 μl of RNA extract with the Platinum One-step qRT-PCR kit (Invitrogen life technologies, Paisley, UK) on an ABI Prism 7500 SDS real-time platform (Applied Biosystems). The following thermal profile was used: a single cycle of reverse transcription for 15 min at 50°C, 2 min at 95°C for reverse transcriptase inactivation and DNA polymerase activation followed by 40 amplification cycles of 15 sec at 95°C and 34 secs at 60°C each (annealing-extension step). Data acquisition occurred at the annealing step of each cycle, and the threshold cycle (Ct) for each sample was calculated by determining the point at which the fluorescence exceeded the threshold limit.

### Statistical Analysis

The percentage detection rate for each pathogen was analysed monthly, and for the overall study period. Binomial 95% confidence intervals were also determined for each detection rate. A chi-squared test was used for any comparisons of two data sets.

Please note that ethical approval was not required for this paper as the samples were collected as part of routine diagnostic work.

## Results

### Detection rate in all samples per month

Examination of the detection rate over the four-month period shows that respiratory pathogens were detected in 27.9% of all samples submitted (95% confidence interval, 26.3-29.5%) (Table 4). Numerous pathogens were detected in the samples. The most commonly detected pathogen was rhinovirus which was detected in 8.9% of all samples tested (95% confidence interval, 7.9-9.9%), and was similarly detected in each of the months examined. Adenovirus was also commonly detected (3.5% (95% confidence interval, 2.9-4.2%). Parainfluenza 3 showed its typical activity. It was detected in 7.9% of all samples collected in April (95% confidence interval, 6-10%) with decreasing activity thereafter (1.9% in July (95% confidence interval, 1.2-2.8%))(p < 0.001)). Interestingly, parainfluenza 1 (a pathogen normally associated with winter activity) was present in an unexpectedly large number of samples during this period (1.9% of all samples overall (95% confidence interval, 1.4-2.4%) and mirrored the increasing activity of H1N1/2009 (from 0.8% in April (95% confidence interval, 0-2%) to 3% in July (95% confidence interval, 2.1-4.1%) (p = 0.0054)). The detection rate for the remaining pathogens was low (< 1.6%) and showed no particular pattern over the months examined.

**Table 4:** Detection rate in all samples per month

	April		May		June		July		Total	
	**Number**	**% (95% CI)**	**Number**	**% (95% CI)**	**Number**	**% (95% CI)**	**Number**	**% (95% CI)**	**Number**	**% (95% CI)**

Influenza B	1	0.2 (0-0.92)			1	0.1 (0-0.7)			2	

Influenza C	2	0.3 (0-1.2)							2	

Parainfluenza 1	5	0.8 (0.-2)	2	0.3 (0-1.1)	18	2.3 (1-3.7)	37	3 (2.1-4.1)	62	1.9 (1.4-2.4)

Parainfluenza 2	0				1	0.1 (0-0.7)	4	0.3 (0-0.8)	5	

Parainfluenza 3	48	7.9 (6-10)	42	6.5 (4.7-8.7)	21	2.7 (1.7-4.1)	23	1.9 (1.2-2.8)	134	4.1 (3.3-4.9)

Parainfluenza 4	0				3	0.4 (0-1.1)	3	0.2 (0-0.7)	6	

Rhinovirus	64	10.6 (8.24-13.3)	52	8 (6-10.4)	58	7.5 (5.7-9.6)	114	9.3 (7.7-11)	288	8.9 (7.9-9.9)

Coronavirus	11	1.8 (0.9-3.2)	9	1.4 (0-2.6)	5	0.6 (0-1.5)	5	0.4 (0-0.1)	30	0.9 (0.6-1.3)

Adenovirus	14	2.3 (1.2-3.9)	25	3.9 (2.5-5.7)	24	3.1 (2-4.6)	50	4.1 (3-5.3)	113	3.5 (2.9-4.2)

Human metapenumovirus	6	1 (0.3-2.2)	15	2.3 (1.3-3.8)	17	2.2 (1-3.5)	15	1.2 (0.6-2)	53	1.6 (1.2-2.1)

Respiratory syncitial virus	21	3.5 (2.1-5.3)	2	0.3 (0-1)	1	0.1 (0-0.7)	2	0.2 (0-0.5)	26	0.8 (0-1.2)

M pneumoniae	2	0.3 (0-1.2)			3	0.4 (0-1.1)	1	0.1 (0-0.5)	6	

Enterovirus	8	1.3 (0.6-2.6)	7	1.1 (0-2.2)	6	0.8 (0-1.6)	6	0.5 (0-1)	27	0.8 (0.5-1.2)

Mixed	20	3.3 (2-5)	9	1.4 (0.6-2.6)	21	2.7 (1.6-4.1)	28	2.3 (1.5-3.3)	78	2.4 (1.9-3)

Overall	202	33.4 (29.6-37.3)	168	26 (22.6-29.5)	198	25.6 (22.5-28.8)	337	27.5 (25-30.1)	905	27.9 (26.3-29.5)

Total	605		647		774		1226		3247	

## Discussion and conclusions

This study highlights the problems of using a clinical algorithm to detect H1N1/2009 during a period of low incidence. We found that clinicians frequently misdiagnosed common respiratory pathogens as H1N1/2009 during the spring/summer outbreak in Scotland. This finding is similar to results found in a recent audit of patients hospitalised with clinically diagnosed H1N1/2009 in infectious disease units in Scotland [8].

The pathogens that were misdiagnosed as H1N1/2009 were, for the most part, those viruses expected to be encountered during the spring/summer months. Although all the pathogens included in the respiratory screen were detected on at least one occasion, rhinovirus was the most commonly detected pathogen. This is not a surprising finding, since rhinovirus is detected all the year round; moreover, it is recognised as a very common cause of the common cold and is increasingly being implicated in more severe clinical syndromes [9]. PF1, PF3, adenovirus and human metapneumovirus were also frequently detected.

An unexpected finding was the frequent detection of parainfluenza 1, a pathogen which is traditionally recognised as a winter pathogen. However, our data shows that there were numerous cases of parainfluenza 1 during April-July 2009, and these mirrored the activity of H1N1/2009. The unexpected summer activity of parainfluenza 1 mirrors that of parainfluenza 4 in 2008, which was also unexpectedly detected during the summer months (data not shown). This suggests that the epidemiology of established viruses, such as parainfluenza 1, should be re-examined in the light of new, more sensitive, molecular assays.

It should be noted that the majority (~70%) of samples submitted to the laboratory were found to be negative by real time rtRT-PCR. By participation in various EQA schemes, the assays used by the laboratory have been shown to be highly sensitive. Consequently, the large number of negative results are unlikely to have been caused by the pathogens already tested for in the screen. However, the respiratory screen does not include an internal control. Consequently, false negatives due to inhibition may have occurred. Nevertheless, the number of samples affected is likely to be small, since a recent in-house audit found that inhibition occurs in ~1% of throat and nasal swabs submitted to our laboratory. Poor sampling may also have led to false negative results. However, as with the case of inhibition, the contribution of this factor is likely to be minimal.

We cannot rule out that the possibility that these samples may have contained other respiratory pathogens not currently included in the respiratory screen (e.g. boca, HKU1 or bacterial respiratory pathogens). In future, the use of a larger testing panel either by further developing the existing service or by using an alternative method (e.g. microarray) would be useful to examine negative samples. Another possibility is that other infective and non-infective agents could have been present. For example, the previously-mentioned audit of patients presenting at infectious disease units found an alternative non-respiratory diagnosis in ~40% of patients initially clinically diagnosed as having H1N1/2009 [8]. Another explanation could be that a number of samples were actually from the worried well, or from asymptomatic contacts of known or suspected cases of H1N1/2009. An audit similar to the one outlined in the publication above [8] would help to clarify this issue.

It should be noted that the samples examined are representative of our coding protocol and may not be representative of the population at large. Consequently, certain patient groups either with or at risk of severe infection may be over-represented in the final data. Whether or not this is the case, the present study shows that numerous respiratory pathogens were being misdiagnosed as H1N1/2009. The misdiagnosis of H1N1/2009 would have had many undesirable consequences. For example, potentially serious conditions may have been wrongly diagnosed as H1N1/2009 [8]. In addition a large number of individuals are likely to have been unnecessarily treated with oseltamivir [10]. This involves unnecessary cost, and would also have exposed individuals to the side effects of oseltamivir [11]. It should further be noted that there could well have been emotional costs in being wrongly labelled as having "swine flu", especially at the early stage of the pandemic, when the severity and outcome of the illness was still largely unknown. In hospitalised patients, unnecessary infection control procedures may have been implemented [10-12]. Disease surveillance may also have been inaccurate, affecting public health measures and leading to increased panic/concern in the general public.

One way to overcome these issues would be to incorporate a near-patient testing component into the algorithm outlined above. This would no doubt reduce the amount of unnecessary treatment and isolation, and would ensure that surveillance data was more accurate. Such a test would need to be very rapid, sensitive, and specific. Although a number of methods have been described, these can be expensive and - in comparison to PCR-based methods - can be insensitive and non-specific [13]. As a result, PCR methods may be required to investigate influenza negative samples and in low prevalence periods, such as the time period examined in this study, PCR may also be required to confirm positive results.

## Competing interests

The authors declare that they have no competing interests.

## Authors' contributions

WFC conceived of the study. RG examined the data and wrote the manuscript. All authors read and approved the final manuscript.

## Pre-publication history

The pre-publication history for this paper can be accessed here:

http://www.biomedcentral.com/1471-2334/11/192/prepub
